# The Role of Biomarkers in Cardio-Oncology

**DOI:** 10.1007/s12265-020-10042-3

**Published:** 2020-07-08

**Authors:** Kajaluxy Ananthan, Alexander R. Lyon

**Affiliations:** 1 Cardio-Oncology Service, Royal Brompton Hospital, Sydney St, Chelsea, London, SW3 6NP UK; 2 National Heart and Lung Institute, Imperial College London, London, UK

**Keywords:** Biomarker, BNP, Brain natriuretic peptide, Cardio-Oncology, Cardiotoxicity, Heart failure, Troponin

## Abstract

In the field of cardio-oncology, it is well recognised that despite the benefits of chemotherapy in treating and possibly curing cancer, it can cause catastrophic damage to bystander tissues resulting in a range of potentially of life-threatening cardiovascular toxicities, and leading to a number of damaging side effects including heart failure and myocardial infarction. Cardiotoxicity is responsible for significant morbidity and mortality in the long-term in oncology patients, specifically due to left ventricular dysfunction. There is increasing emphasis on the early use of biomarkers in order to detect the cardiotoxicity at a stage before it becomes irreversible. The most important markers of cardiac injury are cardiac troponin and natriuretic peptides, whilst markers of inflammation such as interleukin-6, C-reactive protein, myeloperoxidase, Galectin-3, growth differentiation factor-15 are under investigation for their use in detecting cardiotoxicity early. In addition, microRNAs, genome-wide association studies and proteomics are being studied as novel markers of cardiovascular injury or inflammation. The aim of this literature review is to discuss the evidence base behind the use of these biomarkers for the detection of cardiotoxicity.

## Introduction

There is an increasing interest in the use of cardiac biomarkers to guide the management of oncology patients receiving cardiotoxic cancer therapies. Biomarkers offer value to detect early cardiac injury or strain, often preclinical, confirm LV injury and/or dysfunction when imaging provides borderline or inconclusive information, guide treatment strategies including initiation of cardioprotection during ongoing cancer treatment to allow completion of cancer treatment safely and at follow-up to identify patients prior to the development of symptomatic heart failure (HF).

Cardiac biomarkers are generally circulating proteins or molecules which can be detected from blood samples and are markers of cardiotoxicity [1]. In cardio-oncology, cardiac biomarkers provide a way to detect toxicity early, ideally prior to the development of irreversible organ damage, and if elevated, lead to a pathway of care which allows cancer patients to continue their treatment safely. Cardiac biomarkers may be applied at various stages of cancer management, i.e. calculation of baseline risk prior to starting cancer treatment to guide primary prevention treatment, during treatment in monitoring for early toxicity and screening for late side effects in survivors. In the future, there is the potential for using biomarker expression profiles to guide specific choices regarding cardiovascular preventative therapy. The aim of this review is to describe the current role of cardiac biomarkers in oncology patients, in respect of diagnosis, screening and surveillance for the development of cardiotoxicity, both during and after cancer treatment.

### What Is Cardiovascular Toxicity?

The European Society of Cardiology (ESC) [2] in its consensus statement defines cardiovascular (CV) toxicity as ‘any heart injury (functional or structural) related to cancer treatment, taking into consideration chemotherapy, radiotherapy and cancer itself’. CV toxicity can be acute, subacute or delayed presenting many years after chemotherapy or radiotherapy, involving a range of cardiac structures leading to HF, coronary artery disease, valvular heart disease, arrhythmias including cardiac conduction disease and pericardial disease.

Left ventricular injury, dysfunction and/ or HF are the most common cardiotoxicities stemming from cancer therapies. Definition in trials and routine clinical practice vary, which can lead to confusion in management, and currently the role of cardiac biomarkers needs clarification. The ESC Guidelines for the diagnosis and management of HF published in 2016 require an elevated natriuretic peptide level in the diagnosis of HF, but then, defined HF based upon left ventricular ejection fraction (LVEF) into preserved ejection fraction (EF) > 50%, mid-range EF (40–49%) and reduced EF (< 40%) [3]. Therefore, the presence of HF does not depend upon a specific LVEF threshold or value, as LVEF guides treatment options but not the diagnosis. In contrast, clinical oncology trials use the definition of HF as defined by the Common Terminology Criteria for Adverse Events (CTCAE) [4] and the ESMO Practice Guidelines [5], both of which require a change in LVEF, i.e. a decrease of EF by 5% or more to less than 55% in the presence of symptoms of HF or an asymptomatic decrease in EF by 10% or more to less than 55%.

LV dysfunction or HF is a well-recognised complication of anthracyclines and several targeted chemotherapies (Table 1). There is a growing rationale to detect LV dysfunction early, to treat and prevent the development of HF, as well as monitor its progression. Biomarkers are one technology to detect cardiotoxicity early before changes in LVEF, or clinical signs and symptoms of HF have developed. It is important to highlight that biomarkers should not be used in isolation, but rather placed in a comprehensive assessment including determination of cardiac function using imaging, risk factors, clinical symptoms and the context of the cancer (specifically whether it is localised versus metastatic disease and cancer prognosis).

**Table 1:** A comparison amongst the different anthracyclines in terms of relative cardiotoxicity as defined by a decline in left ventricular function and bioequivalent doses [2, 6, 7]

Drug	Relative cardiotoxicity
Doxorubicin rapid infusion	1
Epirubicin	0.8
Daunorubicin	0.6
Idarubicin	0.53
Liposomal anthracyclines	0.5 (for doxorubicin)

Cardiotoxicity surveillance involving the detection of cardiac biomarkers combined with serial imaging may provide the most sensitive strategy to detect early toxicity and guide cardioprotective interventions [8]. Using this combined approach in a cardio-oncology service, cardiologists could support ongoing oncology treatment to completion in 88% of patients referred with cardiotoxicity from their current cancer treatment [9]. For example in a study by Pareek et al. [9], which explored the running of a cardio-oncology service over 5 years, it effectively demonstrated the importance of collaboration between cardiologists and oncologists in order to improve patient outcomes. Another challenge is the role in long-term surveillance for the development of late cardiotoxicity, which is more relevant at present with the increasing cancer survivorship seen. Critical questions include which cancer survivor patient cohorts should be screened, when should screening start, how long patients should be screened for and what biomarkers should be used to detect toxicity from specific oncology treatment regimens.

## Mechanisms of Toxicity

Understanding the pathophysiology of cardiotoxicity is important to guide the selection of appropriate biomarkers for diagnosis and surveillance. Treatments first recognised to cause cardiovascular toxicity were anthracyclines and radiation therapy [10, 11]. Specifically, left ventricular dysfunction is primarily due to anthracycline toxicity, whereas valvular dysfunction and coronary artery disease is due to radiation toxicity [11]. Several targeted cancer therapies cause LV dysfunction and HF including HER2 inhibitors (trastuzumab) [12], RAF-MEK inhibitors [13] and the EGFR inhibitor osimertinib [14]. Other targeted cancer therapies such as vascular endothelial growth factor (VEGF) signalling inhibitors and multi-targeted tyrosine kinase inhibitors have showed a broader spectrum range of CV toxic effects including LV dysfunction but also vascular toxicity, QT prolongation and arrhythmia risk, and in some cases pulmonary hypertension [10, 15]. Therefore, biomarkers for this wider range of CV side effects should be developed for a more comprehensive approach.

**Anthracycline chemotherapy is a cytotoxic form of chemotherapy**, which acts by binding to topoisomerase II and generates reactive oxygen species leading to cell death, which is a common cause of cardiotoxicity [15, 16]. Anthracyclines includes doxorubicin, epirubicin, idarubicin and daunorubicin, and they are used to treat a wide range of cancers including breast cancer, lymphoma, acute leukaemia, sarcomas and a range of paediatric cancers. Approximately 10% of patients experience cardiotoxic side effects stemming from anthracyclines, with some detectable during treatment, others within the first 12 months following treatment and then late presenting cases up to 30 years later [17]. Anthracyclines can cause myocardial toxicity via oxidative stress, mitochondrial dysfunction, which may initially be reversible, before progressing to myocardial injury with apoptosis, necroptosis and cardiomyocyte cell death leading to a troponin leak [6]. Table 1 demonstrates the relative differences in cardiotoxicity amongst the different anthracycline class members.

**Fig. 1:**
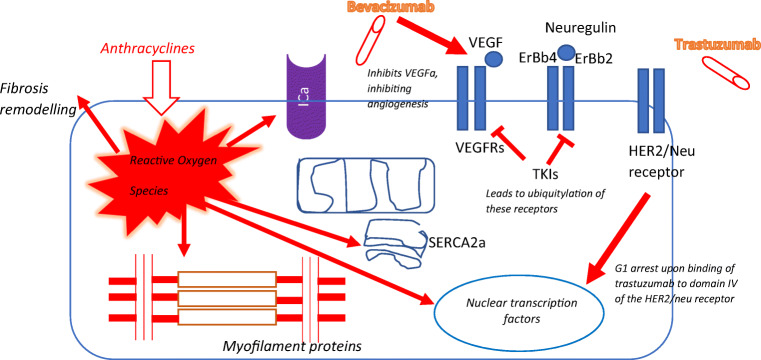
Demonstrates the main mechanism of cardiotoxicity for most cytotoxic chemotherapy. Firstly, anthracycylines have direct mechanisms of damaging DNA resulting in cell cycle arrest leading to either: apopotosis, cell necrosis or cell autophagy. They also generate toxic reactive oxygen species (ROS). ROS trigger off a cascade of events which alters mitochondrial permeability and hence function which moderates calcium signalling and hence myocyte contraction. The mechansim of this invovles the SERCA2a protein which is at the centre of calcium homeostasis. Secondly the receptor tyrosine kinase inhibitors, namely bevacluzumab, trastuzumab, etc. function by inhibiting the activity of their receptor proteins: Vascular Endothelial Growth Factor Receptor (VEGFR), ErBb4/ErBb2 and Her2/Neu receptor resulting in cell cycle arrest in the Growth 1 (G1) phase of the cycle leading eventually to apoptosis, necrosis or cell autophagy. This transition is mediated by p53 (a nuclear DNA-binding phosphoprotein). [22, 155, 156]

Trastuzumab, the humanised monoclonal antibody generated against the HER2/neu receptor, also known as Herceptin®, is known to cause left ventricular dysfunction, which in severe cases can lead to HF. However this HF syndrome is usually, but not always, reversible on cessation of trastuzumab treatment and initiation of HF medications [15, 18]. The mechanisms of cardiotoxicity are complex, and one part is via inhibition of the beneficial and cardioprotective effects of neuregulin-1 signalling via the HER2 receptor in the heart (Fig. 1). Cardiac HER2 signalling is a protective pathway which is upregulated in the hearts under increased stress from pre-existent heart disease or recent trastuzumab treatment [19]. Neuregulin-1 normally acts on the heart via either ErbB4/ErbB4 receptor homodimers or ErbB4/ErbB2 heterodimers to promote protective pathways in the presence of cell stressors [20, 21]. Trastuzumab causes functional abnormalities rather than myocyte necrosis or apoptosis, unless concomitantly delivered with other chemotherapeutic agents [22, 23]. Hence, this suggests that it directly causes stress rather than myocyte injury, but in patients with actived anthracycline-initiated apoptosis, it may exacerbate the injury and myocyte cell death by inhibiting the antiapoptotic pathways. Other chemotherapeutic agents responsible for reversible cardiotoxicity include VEGF inhibitors [22].

VEGF inhibitors (VEGFi, e.g. pazopanib, sorafenib, sunitinib) [24] and BCR-ABL tyrosine kinase inhibitors [15] are examples of anti-cancer therapies which can cause both direct LV systolic dysfunction and indirect LV dysfunction via coronary artery disease including acute myocardial infarction and hypertension [25]. Other CV adverse effects include thrombotic events, QTc interval prolongation and HF [26–29].

Schutz et al. calculated that the relative risk of mortality was 2.23 (95% CI, 1.12–4.44) in a meta-analysis of 4679 patients treated with sunitinib, sorafenib or pazopanib [30]. VEGF inhibitor–related CV toxicity is usually reversible when the VEGFi is stopped and with appropriate CV treatment [22]. The clinical challenge in these cases is that VEGFis are a key component of treatment of a range of metastatic cancers, and therefore efforts must be made to control the CV toxicity in order to continue treatment safely.

VEGF-related hypertension occurs via various mechanisms, including impaired endothelial function secondary to decreased nitric oxide availability, increased vascular tone and decreased microvascular density [31]. Renal dysfunction due to thrombotic microangiopathy is an important factor leading to hypertension especially with bevacizumab [32]. The development of hypertension with VEGFis is associated with improved survival in patients with metastatic renal cancer. Rini et al. [33] showed an improved survival of 30.9 months (95% CI = 27.9–33.7 months) in patients who developed hypertension with bevacizumab compared with 7.2 months (95% CI = 5.6–10.7 months) in patients who did not. Therefore, although the development of hypertension is a complication, it is also a marker of efficacy.

Proposed mechanisms for the development of thrombotic events include disruption of endothelial cell integrity [27], platelet aggregation and degranulation [34], which are all considered reversible. However, thrombotic events and myocardial infarction lead to increased mortality which has been confirmed in various meta-analysis [35]. Despite all these potentially serious CV toxicities from VEGF inhibitors, it is important to remember that there is improved survival from cancer and overall survival on patients on VEGF inhibitors. The role of cardiac biomarkers in VEGF inhibitor–related CV toxicities is limited, and the only biomarkers currently available with growing evidence are natriuretic peptides (NPs). Nevertheless, it has been shown that asymptomatic cardiotoxicity is increasingly more common, but was only detected in 43 patients (27%) by either an increase in NPs or a decline in LVEF [36]. Morever, there is no specific link between an elevation in specific biomarkers and the biological mechanisms behind causing cardiotoxicity. Biomarkers which could detect early LV dysfunction more specifically than NPs, identify VEGFi-related hypertension early on, or predict arterial thrombosis could lead to more specific treatments to prevent clinically significant CV toxicities and keep patients on their effective VEGFi for longer [33, 35, 37].

The immune checkpoint inhibitors (ICIs) are a new class of cancer therapies, which activate the immune system to target and destroy cancer cells. ICI target receptors and signalling pathways involved in immune escape of cancer cells include cytotoxic T lymphocyte–associated protein 4 receptor (CTLA4), programmed cell death protein-1 (PD-1) and programmed cell death protein ligand-1 (PD-L1) [38]. Despite being shown to enhance clinical outcomes in cancer patients, ICIs can also cause a range of cardiovascular toxicities [39]. Most of the CV toxicities from ICI treatments are immune-mediated, and the most common and most serious is autoimmune T-lymphocytic myocarditis. In addition, ICIs can cause complete heart block, acute coronary syndromes secondary to both atherosclerotic plaque rupture and autoimmune vasculitis, pericarditis and a new emerging syndrome of non-inflammatory left ventricular dysfunction [38]. More studies are required to phenotype the spectrum of ICI-mediated CV toxicities and the role of biomarkers in diagnosis, surveillance and monitoring response to treatment agents [40].

Chimeric antigen receptor therapy (CAR-T therapy) is a novel form of immune therapy where T cells are genetically engineered to target tumour cells. CAR-T cell therapy is used in the treatment of various haematological and solid cancers; the main side effect is a cytokine release storm (CRS) leading to a systemic inflammatory response syndrome with multiorgan failure including cardiotoxicity. There is also an increased risk of arrhythmias, namely SVT and atrial fibrillation [41]. A study assessing cardiovascular complications in 93 children treated with CAR-T therapy for leukaemia reported that there were 21 cases of hypotension requiring inotropic support with onset at a mean of 4.6 days [42]. In this group, 41% had systolic dysfunction on echocardiogram, whilst 7% had new cardiac dysfunction maintained until discharge, this was persistent at 6 months in 1 patient only [42]. In a recent report of 137 adult cancer patients receiving CAR-T, the evidence of new CV events occurred in 31% of the patients with grade > 2 CRS including decompensated HF, arrhythmias and CV death [43].

## Current Methodology for Diagnosis of Cardiotoxicity

The American Society of Echocardiography and the European Association of Cardiovascular Imaging have defined one feature of cardiotoxicity as the reduction in LVEF > 10% resulting in a value < 53% (normal limit if 2-Dimensional echocardiography is used) [44]. The European Society of Cardiology 2016 have stated that echocardiography (3-Dimensional if endocardial border is clear or 2-Dimensional with biplane Simpson method) is the most optimal choice for detecting cardiotoxicity leading to left ventricular dysfunction at all stages of chemotherapy, with cardiotoxicity defined as a decrease in LVEF > 10% to reach a value below the normal limit for ejection fraction [2]. The frequency of assessment needed to detect cardiotoxicity is determined by the underlying cancer treated, the cardiotoxic cancer therapy and planned duration, type, total dose and baseline cardiovascular risk in all haematology and oncology patients. It is increasingly recognised that diagnosing cardiotoxicity based on symptoms alone or waiting for a decline in ejection fraction which causes symptoms is too late, and it means that the degree of myocyte damage is no longer reversible. Also, this method does not detect cardiotoxicity manifesting structurally such as myocarditis.

A prospective study [17] involving a large cohort of patient showed that regular echocardiography allowed identification of cardiotoxicity in 98% of patients in the first year of treatment, such that with treatment using ACE inhibitors and beta blockers, 82% of patients were able to recover on follow-up and achieve a normal EF. However, in 71% patients, the EF achieved with this medical treatment was below their value prior to chemotherapy. This emphasises the importance of earlier detection of cardiotoxicity before a decline in EF, which paves the importance of biomarkers as a tool for early detection of cardiotoxicity in current cardio-oncology practice.

## Cardiac Troponin

Cardiac troponins (cTn) (both troponin I and troponin T) are the gold standard biomarkers for the detection of cardiac injury and cardiomyocyte necrosis, and the most extensively used biomarker to detect cardiac toxicity [45, 46]. They have high specificity for cardiac injury, but also with the advent of high sensitive assays means that it is possible to detect small amounts of myocyte damage and help provide treatments to minimise cardiotoxicity prior to the development of irreversible LV dysfunction [47]. The importance of monitoring troponin to detect cardiotoxicity has been demonstrated mainly from studies of cancer patients receiving chemotherapy, mainly anthracycline [48]. A persistent elevation of troponin I is associated with a greater degree of LV dysfunction and a higher incidence of cardiac events compared with transient elevations in the troponin levels [47]. Troponin T is generally considered less useful as a marker as studies have shown that it is not associated with myocardial infarction or coronary heart disease, but instead is more closely linked to the risk of death which is not directly attributed to cardovascular disease [49].

### Anthracyclines

The importance of troponin as a marker of anthracycline-induced cardiotoxicity was first demonstrated in animal models. Clinical studies in both children and adults have shown that elevated troponin levels during chemotherapy is an early marker of increased risk of left ventricular dysfunction [46–48, 50–67]. The largest study to date involved 703 patients with cancer where troponin I was measured using a conventional assay (defined as an assay cut-off of above 80 ng/L representing a significance elevation in troponin I) before chemotherapy, within 3 days of commencing chemotherapy and after 1 month [47]. Troponin I was in the normal range in 70% (495 patients), and in the remaining 208 patients increased at 3 days only in 145 patients and increased in both early and late stages in 63 patients. Patients without troponin I elevations had no significant reduction in LV function and had a good prognosis with an incidence of 1% of cardiac events at 3-year follow-up. On the other hand, elevation of troponin I was associated with a higher incidence of adverse cardiovascular events. Specifically, the maintenance of troponin I elevation after 1 month was associated with greater LV impairment and higher incidence of cardiovascular events compared with an isolated elevation of troponin I at only 3 days (84% vs 37%;*p* < 0.001) [47]. Similarly, the incidence of adverse events was greater in the group who had a persistent rise in the troponin levels during anthracycline chemotherapy.

Another study involving 204 patients with malignancies requiring anthracyclines involved measuring troponin for every cycle of high-dose chemotherapy [46]. In total 65 patients 32% had elevated troponin (> 50 ng/L). This was linked to a greater reduction in LVEF on long-term follow-up such that when LVEF <30%, 100% of patients had troponin >50ng/L and the higher the troponin the greater the reduction in LVEF. Both studies, hence, validate the importance of using conventional troponin for surveillance post-chemotherapy as both a sensitive and reproducible marker of myocardial dysfunction, prior to development of HF. There is also increasing data emerging from survivors of childhood cancers which shows that cTnI and hs-TnI were not consistently elevated in patients who developed LV dysfunction on imaging [68–70]. Hence, these studies demonstrate that although troponin is a sensitive marker during and early after chemotherapy, it is not a sensitive marker for ongoing surveillance of subclinical cardiotoxicity post-chemotherapy, when compared to other biomarkers such as NPs.

### Trastuzumab

Troponin I has a negative predictive value of 99% which helps reliably identify patients who do not need regular follow-up [47]. The role of troponin as a marker of cardiotoxicity with other cancer therapy regimens apart from the anthracyclines is still under research [48, 50, 52, 56, 57, 59]. In breast cancer patients treated with trastuzumab, 16 patients who developed LV dysfunction did not demonstrate an increase in troponin [48]. LV dysfunction quickly recovered on cessation of treatment, hence this could represent myocardial sensence induced by trastuzumab instead of myocaridal toxicity. This same study involving 251 trastuzumab-treated patients showed than an increase in troponin in 36 patients (14%) predicted LV dysfunction and poor prognosis, and hence demonstrates its specificity for use in clinical practice [48].

The largest study of cardiac troponin is the HERA trial cardiac biomarker substudy of 533 women receiving trastuzumab for HER2+ breast cancer [71]. They reported an elevated cardiac troponin post-anthracycline treatment but before trastuzumab (baseline pre trastuzumab) and that this baseline troponin predicted which women developed future trastuzumab-induced cardiotoxicity as signified by a decline in LVEF (hazard ratio 3.57–4.52 vs no troponin rise), whereas troponin surveillance during trastuzumab was not sensitive. In line with the HERA trial substudy, several other studies also reported that surveillance with cardiac troponin during trastuzumab treatment did not predict LV dysfunction [72–74]. This could be related to the different preceding chemotherapy regimens (anthracyclines vs non-anthracyclines), the variation in the sampling time, the different troponin assays used and the length of follow-up [51]. For example, it has been shown that exposure to doxorubicin in childhood can lead to increased risk of morbidity and mortality [75, 76] of up to 3.1-fold in 30-year-old survivors [77].

### Immunotherapy

Cancer treatment has greatly advanced with the widespread use of immune checkpoint inhibitors [78]. Nevertheless, their increasing use is not without problems, specifically immune checkpoint inhibitor (ICI)–mediated myocarditis [79–82]. ICI-mediated myocarditis can be detected through an increase in serum troponin, which is one of the main diagnostic criteria for diagnosis in general cardiology. However, in one of the larger published cohorts of ICI myocarditis, only 94% of patients had an elevated troponin at presentation [79]. Therefore, 6% of the cases in this cohort had ICI-mediated myocarditis diagnosed clinically with normal cardiac troponin levels. This, therefore, demonstrates that biopsy-proven, troponin-negative ICI-mediated myocarditis can occur. The paper from Mahmood et al. [79] also highlighted the increased mortality if patients are discharged from hospital with myocarditis still active and not fully suppressed. In this cohort, the patients with cardiac troponin at discharge that was greater than 1.5 ng/mL had a fourfold higher risk major adverse cardiac events (MACE) at follow-up. Therefore in addition to diagnosis, serial troponin measurements are required to monitor response to immunosuppressant treatment and timing of when patients with ICI myocarditis are safe to discharge home on oral steroid therapy. A recent paper defined the criteria for ICI-related myocarditis [80]. The role of troponin surveillance has been proposed and requires prospective research studies for validation, at least for patients considered at higher risk of myocarditis and CV complications [80, 81]. If applied, it is recommended that troponin measurements at baseline and weekly during cycles 1–3 can detect this myocarditis-related toxicity early [82].

### CAR-T Cell Therapies

The study of 137 adult cancer patients receiving CAR-T reported that cardiac troponin was a sensitive biomarker for the detection of CV toxicities [43]. Cardiac troponin was found to be elevated in 29 of 53 tested patients (54%) and was elevated in 95% of patients who subsequently developed clinical CV toxicity. Therefore, it appears that an inflammatory CAR-T-mediated myocarditis may occur in some patients as part of their cytokine release storm [83], and occurs early (with a median of 5 days after treatment) [43]. Hence, a short period of cardiac troponin surveillance may be warranted and should be studied prospectively in larger cohorts.

## Natriuretic Peptides

Two NPs, i.e. brain natriuretic peptide (BNP) and N-terminal pro-B type natriuretic peptide (NT-proBNP) [8, 52, 84–86] are simulated to be secreted by cardiomyocytes from increased transmural tension and neurohormonal stimulation (notably by noradrenaline and angiotensin II). BNP is derived from cleavage of pre-proBNP leading to BNP and the biologically inactive N-terminal containing fragment (NT-proBNP). NT-proBNP has also been used in it own right as a biomarker for detection of HF [87, 88]. Hence, NPs are important biomarker of pressure overload and myocardial stretch [89, 90] and, their ability to detect haemodynamic stress makes them an important maker of cardiotoxicity, as well as for long-term surveillance in the management of heart failure. NPs may also be used to detect acute cardiotoxicity as levels increase within 24 h of exposure to anthracycline chemotherapy [91, 92]. NPs are also an important screening tool for HF in patients presenting with dyspnoea on cancer therapy, but it has been shown that a cut-off value of 100 ng/L for NT-proBNP has a high sensitivity (0.92), but low specificity (0.5) for cardiotoxicity as there are other causes of increased NPs including, for example atrial fibrillation and valvular heart disease [93].

Several studies have analysed the role of NPs in various oncology populations. One study measured NP in an unselected cohort of 600 successive newly diagnosed cancer patients to their large oncology service. An elevated baseline NT-proBNP pre-oncology treatment was a signifcant predictor of mortality risk: hazard ratio 1.54 (95% CI 1.24 to 1.90, p<0.001) with a Kaplan–Meier survival rate of 67% versus 49% (in patients with normal NT-proBNP, p<0.001) at a median of 25 months follow-up [94].

Most studies of NPs have been in patients receiving anthracyclines or anthracyclines and trastuzumab; treatments which both cause LV dysfunction and HF. One study evaluating NT-proBNP in 52 patients treated with high-dose anthracyclines with normal NT-proBNP at baseline, three different patterns of elevation of pro-BNP have been identified [95]: 17 patients had elevated NT-proBNP immediately after starting treatment which remained elevated at 72 hours [160 (95) ng/L at baseline; 1163 (936) ng/L after 72 h; P <0.00038], in 19 patients NT-proBNP only increased after 12-36 hours after treatment and decreased back to baseline after 72 hours [120 (107) ng/L at baseline; 185 (101) ng/L after 72 h; P = 0.002], and in the final group NT-proBNP decreased from baseline after 72 hours [90 (95) ng/L at baseline; 39 (19) ng/L after 72 h; P = 0.04]. In patients with high NT-proBNP at 1-year follow-up, there was a significant reduction in LVEF, and there was a strong correlation between the level of NT-proBNP at 72 h and the degree of reduction of EF at 12-month follow-up as well as other markers of systolic and diastolic dysfunction including: change in ratio of peak early to peak late flow velocities ( E/A) from >1 to <1, E-wave deceleration time and isolvolumetric relaxation time. Therefore, measurement of NPs not only helps identify patients who will develop cardiotoxicity, but may also help determine the degree of cardiac dysfunction.

Similar evidence also exists for BNPs as a marker of cardiotoxicty. A single-centre study of 109 cancer patients undergoing treatment with anthracyclines showed that 10.1% of patients experienced a cardiac event (namely LV dysfunction, symptomatic HF, arrhythmia, sudden cardiac death or ACS), and all of these patients had a BNP > 100 ng/L prior to the event [92]. This supports the strategy of regular BNP measurements as well as imaging to detect cardiotoxicity. However this study highlighted that not all these patients ended up developing LV dysfunction. On the other hand another study involving children treated with doxorubicin, BNP elevation was predictive of development of cardiomyopathy as signified by LV dysfunction (p<0.01) when compared to healthy patients as the control [96]. Similarly, in the HERA breast cancer trial of trastuzumab, the cardiac biomarker substudy reported that new rises in NT-proBNP during trastuzumab treatment were most predictive of a subsequent reduction in LV function on echocardiographic surveillance, although it was a trend which did not reach statistical significance [71]. However there remains disparity with the results, for example another study involving 43 patients with left-sided breast cancer after radiotherapy, serial BNP measurements up to 12 months post-therapy showed that small BNP elevations were predictive for the development of radiotherapy-related cardiovascular events, although none of the patients developed evidence of LV dysfunction [97].

In patients receiving ICIs, the main application of NPs was in the diagnosis of new LV dysfunction. This can be in the context of ICI-mediated myocarditis [79], although NPs are raised in only two-thirds of cases with myocarditis. The main role of NPs in our clinical practice is to diagnose non-inflammatory LV, dysfunction as this new and emerging clinical entity can be partly differentiated by the rise of NPs with a normal serial cardiac troponin levels, ideally measured with a high sensitive assay. The diagnosis also requires clinical and imaging evidence of new LV dysfunction and absence of myocardial inflammation on cardiac imaging and/ or endomyocardial biopsy.

Proteasome inhibitors (PIs) are an effective treatment of multiple myeloma (MM), and 3 are currently licensed (bortezomib, carfilzomib and ixazomib). PIs have various cardiovascular toxicities, and in particular, the most potent PI carfilzomib can cause HF, acute coronary syndromes, arrhythmias and sudden death. Cornell and colleagues [98] reported the CV complications in 95 adult MM patients treated with carfilzomib (*n* = 65) or bortezomib (*n* = 30). Clinical CV complications, called CV adverse events (CVAEs), was 50% in carfilzomib-treated and 17% in bortezomib-treated MM patients, including HF, ACS, arrhythmia, pulmonary arterial hypertension, hypertension and venous thromboembolism. Elevated baseline NPs before treatment was associated with a 10.8-fold increased risk of a CVAE compared with MM patients with a normal baseline NPs. A new rise in NPs in the carfilzomib-treated patients during treatment occurred predominantly in the first 6 months of treatment, but specifically it has been shown that the early rise taking within the first 3 weeks of carfilzomib treatment was associated with a 36-fold increased risk of CVAEs (95% CI, 4.4 to 296; *p* < 0.001 vs no rise). CVAEs were associated with interruption of PI and worse overall survival (median 18.1 months for patients with CVAE vs not reached in the MM patients without a CVAE), with 88% of deaths due to MM disease progression. Therefore, measurement of NPs at baseline and early during treatment may help identify higher risk patients for closer surveillance and implementation of cardioprotective treatments and intense control of CV risk factors.

Limitations in all the studies assessing the role of NPs in cancer patients are small study sizes, retrospective design and lack of reference ranges, cohorts are small and unmatched cohorts between different studies, different cancer types with different chemotherapy regimens, limited follow-up, variable defined endpoints, variable cut-off values for BNP and timepoints when the BNP was measured. It is also recognised that in elderly patients and females, high levels of NPs can be normal [99, 100]. Similarly, renal dysfunction can also increase levels of NPs [101], whereas obesity is associated with lower levels of NPs. Finally, inflammation secondary to cancer has also been shown in some studies to cause elevation of NPs [102].

### Do Troponin and Natriuretic Peptides Convey Different Information?

Troponin as a biomarker defines cardiomyocyte injury [65, 103, 104] (Table 2), whereas BNP is a marker of increased myocardial strain (Table 3) [65, 90]. NPs are excellent markers of long-term cardiovascular dysfunction in asymptomatic patients [138]. Other biomarkers provide information on acute injury but may not remain elevated in the long-term.

**Table 2:** This table summarises the studies demonstrating the value of either troponin I or troponin T to detect cardiotoxicity including the type of cancer treatment, the characteristics of the cohort studied, percentage positive troponin at both baseline and after treatment, idenitification of the mechanisms involved to lead to cardiotoxicity, and the assay type and cut-off values used

Treatment type	Reference	Patient cohort and age	Gender (male:female ratio)	Percentage of troponin positive (%) after treatment	Percentage of troponin positive (%) before treatment	Mean troponin values (after treatment)	Troponin cut-off	Troponin assay	Mechanism of cardiotoxicity
Anthracycline	Cardinale (2000) [46]	Advanced cancer high-dose chemo, 45 ± 10 years	39:165	65/204 (32%)	0	1000±500ng/L	500 ng/L, Troponin I	Stratus II TnI	Direct cardiotoxicity
Anthracycline	Cardinale (2002) [105]	Breast CA with HDC, 46 ± 11 years	211 F	70/211 (33%)	0	900± 500ng/L	500 ng/L, Troponin I	Stratus II TnI	Direct cardiotoxicity
Anthracycline	Cardinale (2004) [47]	Advanced cancer with high-dose chemo, 47 ± 12 years	216:487	208/703 (30%)	0	160 ± 240ng/L	80 ng/L, Troponin I	Stratus CS Tn I	Direct cardiotoxicity
Anthracycline	Lipschultz (2004) [106]	ALL children Dox vs Dex + Dexraz RCT, 7.4 years old on average	120:86	55/158 (35%).	12/119 (10%)	Tn + 50% Tn++32% Dex 21%, 10%	10ng/L	Roche ElecsysTnT	Direct cardiotoxicity
Anthracycline	Auner (2003) [62]	Haematological adults, 58 years old	42:36	12/78 (15.4%) Delta EF > 10%	3/78 (3.8%)	Med 40ng/L; [33–74, 78–82, 84–86, 91–102, 107–135]	30 ng/L, Troponin T	Roche ElecIII TnT	Direct cardiotoxicity
Anthracycline	Sandri (2003) [63]	Advanced cancer high-dose chemo, 47 ± 11 years	42:137	(31.8%) Delta EF − 18%	1%	540Troponin pos 0.63 ±0.54μg/L Troponin neg 0.039 ±0.019μg/L	80 ng/L, Troponin I	Stratus II CS Tn I	Direct cardiotoxicity
Anthracycline	Specchia (2005) [64]	79 patients diagnosed with acute leukaemia, ages ranging from 14 to 79 years	52:27	11–12%	N/A	Not stated	0.15 ng/mL, Troponin I	Becton Dickinson	Direct cardiotoxicity
Anthracycline	Kilickap (2005) [65]	Advanced haem CA with high-dose chemo, 44 years old	20:21	14/41 (the elevated but exceeded the cut off in only 1 patient)	*N* = 1 (0.016 ng/L)	Not stated	0.10ng/mL up per limit of normal troponin consider ed eelvated if >0.010ng/mL	Roche ElecIIITnT	Direct cardiotoxicity
Anthracycline	Sawaya (2011) [50]	43 females with HER-2 positive breast cancer, age 48	No males	12/43	N/A	Not stated	0.015 μg/L, Troponin I	Siemens Healthcare Diagnostics, Tn I	Direct cardiotoxicity
Anthracycline	Sawaya (2012) [52]	81 females with HER-2-positive breast cancer treated with anthracyclines followed by taxanes and trastuzumab, age 50 years	No males	26/81(32.1%)	N/A	32 pg/mL (10-56pg/mL)	30 pg/ml, Troponin I	Siemens Healthcare Diagnostics, Tn I	Direct cardiotoxicity
Anthracycline	Drafts (2013) [53]	53 participants, average age 50 years, 58% female, 83% white treated with doxorubicin or daunorubicin	42%:58%	Not stated, only states 26% had Troponin I value between 0.06-1ng/mL	N/A	0.04 ± 0.01 ng/mL	0.06 ng/mL, Troponin I	Beckman Coulter	Direct cardiotoxicity
Anthracycline	Mavinkurve-Groothuis (2013) [55]	120 children diagnosed with ALL	77:43	11%at 10 weeks, and 13.3% at 1 year	0%	0.01–0.04 ng/mL	0.01 ng/mL, Troponin T	Roche Diagnostics	Direct cardiotoxicity
Anthracycline	Haney (2013) [136]	22 female breast cancer patients, age not stated	No males	41% (9/22)	N/A	Peak 60 ng/L cycle 6:50% Troponin positive, all positive results between cycles 4-6	12 ng/L	Roche TnT	Direct cardiotoxicity
Anthracycline	Katsurada (2014) [137]	19 females with HER2 positive breat cancer and about to receive adjuvant chemotherapy with trastuzumab, anthroacyclines or taxanes	No males, 2 groups based on BMI, Group R- BMI 25 ±3kg/m2 (N=9), Group N -BMI 22±2kg/m2 (N=10)	Not stated how many over cut-off values, but appears at all timepoints significantly elevated compared to baseline in both groups	N/A	TROPONIN T: In Group R (N=9) Troponin T: 11 ± 7.8pg/mL at 6 months, (p<0.01), In Group N (N=10) Troponin T: 7± 5.8 pg/mL at 3 months, (p<0.05), 4 ± 1.4TROPONIN I: In group R (N=9) Troponin I: 19.7 ±17.3pg/mL at 3 months, (p<0.001), In Group N (N=10) Troponin I: 14.1 ± 7.0 pg/mL at 3 months, (p<0.001)	14 pg/mL, Troponin T; 40pg/mL, Troponin I	Roche hs-TnT	Direct cardiotoxicity
Anthracycline	Ky (2014) [56]	78 HER-2-positive breast cancer patients undertaking adjuvant anthracycline regimen followed by taxanes and trastuzumab, median age 50 years	No males	Not stated	N/A	13.9 (2.6 to 31.6) μg/L elevation	6.2 ± 13.7 μg/L, Troponin I	Siemens Healthcare Diagnostics	Direct cardiotoxicity
Anthracycline	Putt (2015) [57]	78 women planned for adjuvant therapy with an anthracycline-containing regimen followed by taxanes and trastuzumab, mean age 49 years old	No males	Not stated	N/A	3 months: 9.9 ng/L 6 months: 12.0 ng/L 9 months: 6.8 ng/L, 12 months: 4.2 ng/L, 15 months: 3.9 ng/L	3 ng/L, Troponin I	Siemens Healthcare Diagnostics	Direct cardiotoxicity
Anthracycline	Olivieri (2017) [58]	Lymphoma patients undergoing first line anthracycline or liposomal anthracycline treatment, mean age 60 years old	99 patients, 56:43	42/99 (42%)	4/99 (4%)	Not stated	30 ng/L, Troponin I	Not stated	Direct cardiotoxicity
Anthracycline	Kitayama (2017) [59]	40 females with breast cancer undergoing treatment with anthracycline or trastuzumab, aged 55-57 years	No males	4/40 (10%)	0%	Troponin T 0.044 ± 0.0109 ng/mL	This paper evaluated threshold called high sensitivity troponin T integration value above baseline set at 0.07ng months/mL as 100% sensitive and specific for the detection of cardiotoxicity, from this troponin T 0.04ng/mL derived as threshold	EcLusys high-sensitivity Troponin T, Roche Diagnostics	Direct cardiotoxicity
Anthracycline	Shafi (2017) [60]	82 females with newly diagnosed breast cancer, median age 47 years	No males	18/82(22%)	0%	Not mentioned, Troponin I	Not mentioned, Troponin I	Stratus II	Direct cardiotoxicity
Trastuzumab	Cardinale (2010) [48]	251 women (mean age, 50 ± 10 years) with breast cancer undergoing treatment with trastuzumab	No males	36/251 (14.3%)	7/25 1 (2.8%)	0.31 ± 0.45 ng/mL	0.08 ng/mL, Troponin I	Stratus CS, TnI	Direct cardiotoxicity
Trastuzumab	Zardavas (2017) [71]	452 females with HER-2-positive breast cancer after neoadjuvant chemotherapy, median 50 years	No males	Not stated	Troponin I: 56/412 (13.6%), Troponin T: 101/407= 24.8%	Not stated	Troponin I 40 ng/L, Troponin T 14 ng /L	cTnI Ultra assay Siemens Healthcare Diagnostics, cTnT-hs assay Elecsys platform (Roche Diagnostics)	Direct cardiotoxicity
Immune checkpoint inhibitors	Mahmood (2018) [79]	140 cases derived from 8-centre institutional registry, mean age 65 years	97:43	Elevated serum troponin T in 94% patients presenting with myocarditis.	N/A	2.68 (0.24–7.63) ng/mL troponin T	Not stated, Troponin T	4th generation Troponin T assay	Immune mechanisms
Chimeric antigen receptor therapy (CAR-T therapy)	Alvi (2019) [43]	137 patients who received CAR-T therapy, median age 62 years	93:44 (68%:32%)	29/137 (21.2%)	Not stated	63 ng/L (34 - 110 ng/l)	0.03 ng/ml, Troponin T	Not stated	Indirect effect of cytokine release storm causing myocarditis

**Table 3:** Role of BNP as a biomarker in the detection of cardiovascular toxicity of cancer therapeutics and immune therapies. This table summarises the studies demonstrating the value of BNP to detect cardiotoxicity including the type of cancer treatment, the characteristics of the cohort studied, percentage positive troponin at both baseline and after treatment, idenitification of the mechanisms involved to lead to cardiotoxicity, and the assay type and cut-off values used

Treatment type	Reference	Patient cohort	Gender (male:female ratio)	Percentage of BNP positive (%) after treatment	Percentage of BNP positive (%) before treatment	BNP values	BNP cut-off (pg/mL)	BNP assay	Mechanism of cardiotoxicity
Anthracycline	Daugaard (2005) [84]	107 patients, mean age 55 years with disseminated malignant disease treated with either doxorubicin or epirubicin	22:85	Not stated	N/A	Not stated	0.5 pmol/L	Radio-immunoassay using rabbit antibodies and Sep-Pak C18 cartridges	Direct cardiotoxicity
Anthracycline	Lenihan (2016) [92]	109 patients starting a new course of chemotherapy, median age 56 years	52:57 48%:52%	Not stated	1/109 (0.9%)	Not stated	100 pg/mL	Not stated	Direct cardiotoxicity
Anthracycline	Hayakawa (2001) [96]	34 patients who have received doxorubicin-containing chemotherapy	18:16	8/34 (23.5%)	N/A	Not stated	Not stated	Not stated	Direct cardiotoxicity
Anthracycline	Lee (2008) [66]	86 patients with mostly malignant lymphoma, mean age 48.5 years	49:37	Not stated	N/A	305.8 pg/mL	100 pg/mL	Triage®	Direct cardiotoxicity
Anthracycline	Palumbo (2016) [97]	43 female patients with left-sided breast cancer, mean age 63 years	No males	Not stated	Not stated	23.96 ± 15.08pg/mL at 1 month	6.7 ± 18.6 pg/mL	Shinoria-BNP	Direct cardiotoxicity
Anthracycline	Kitayama (2017) [59]	40 female breast cancer undergoing treatment with anthracycline or trastuzumab, aged 55–57 years	No males	No significant change from baseline	Not applicable	20 ± 6 pg/mL	18 ± 2.2 pg/mL (*p* = 0.7, not statistically significant change from baseline in BNP value)	BNP-JP Architect	Direct cardiotoxicity
Anthracycline	Sawaya (2011) [50]	43 females with HER-2-positive breast cancer, age 48	No males	No significant change from baseline	N/A	Not stated	NT-proBNP 125 pg/mL	Siemens Healthcare Diagnostics	Direct cardiotoxicity
Anthracycline	Mavinkurve-Groothuis (2013) [56]	120 children	Not stated	20% (8/41)	26% (12/46)	Not stated	age-dependent reference vaues 97.5% percentile	Roche Diagnostics	Direct cardiotoxicity
Trastuzumab	Zardavas (2017) [55]	102 females with HER-2-positive breast cancer after neoadjuvant chemotherapy, median 50 years	No males	Not stated	Not applicable	mean absolute increase in NT-pro BNP of 177 ng/L	Not stated	Elecsys NT-proBNP assay	Direct cardiotoxicity
Proteasome inhibitors	Cornell (2019) [98]	95 patients with relapsed MM, (defined by International Myeloma Working Group) treated with carfilzomib- or bortezomib-based therapy, median age 66 years	63:32	Not stated	N/A	Median BNP rise 165 pg/mL, median NT-pro BNP 5925 pg/mL	BNP 100 pg/mL, NT-proBNP 125 pg/mL	Not stated	Direct cardiotoxicity
Vascular endothelial growth factor inhibitors	Catino (2018) [37]	84 patients with metastatic renal cell carcinoma about to start sunitinib, median age 62.5 years	56:28	Not stated	N/A	Not stated	cut off not mentioned but BNP 31.7 pg/mL at baseline	Singulex single molecule counting assay	Direct cardiotoxicity

## Role of Biomarkers to Detect Shared Risk Factors for Heart Failure or Cancer

Evidence is emerging about a common underlying pathophysiology between cardiovascular disease and cancer [107]. Inflammation is a common mediator of both processes, and hyperglycaemia, hyperinsulinaemia and IGF-1 are also important [108]. In this way, markers of inflammation are increasingly recognised as important in detection of cardiotoxicity.

### Inflammatory Mediators

Interleukin-6 has been shown to be associated with increased risk of cardiovascular events (MACE, MI and cardiovascular death) as well as all-cause mortality and increased risk of cancer [109]. IL-6 is considered an important inflammatory marker important in the process of atherosclerosis. Its effects are mediated by downstream inflammatory proteins, namely C-reactive protein (CRP) [110].

CRP is an acute phase reactant produced by hepatocytes due to simulation by interleukin-6 produced by macrophages and T cells [110]. Elevated hsCRP is associated with decreased LVEF in patients with varying cardiovascular disease ranging from myocardial infarction and HF [110]. A study involving 54 women treated with trastuzumab showed that hsCRP > 3 mg/L was 92.9% sensitive and 45.7% specific for LVEF reduction which is clinically significant [111]. Peak levels of hsCRP were detected after a median of 78 days before cardiotoxicity became evident as quantified by a decrease in LVEF. However, other studies failed to show a conclusive link between elevated CRP and cardiotoxicity [8, 139]. A specific use of CRP may lie with T cell therapies, namely Chimeric Antigen Receptor (CAR) T cells where daily measurements of CRP is essential to detect cytokine release storm, which may present as severe hypotension, LV dysfunction, arrhythmia and cardiac arrest [56, 112]. Nevertheless, the limitation of hsCRP is that is has a low specificity when elevated of 45.7% and a positive predictive value for the detection of a decline in LVEF of 40.6%. Hence due to the risks of achieving false positive values, a high level does not confirm developement of LV dysfunction [111].

There is increased recognition of the overlap between innate immune and inflammatory pathways which are key in cardiac remodelling [113, 114]. The central mediators are expressed in both cardiomyocytes and fibroblasts. When the adaptive immune system is activated, the activation of antigen presenting cells by T cells is key to healing after a myocardial infarction and cardiac remodelling pathways [115, 116]. This immunological, pro-inflammatory response which in turn is important in the pathophysiology of HF also has an impact on the physiology of distant organs including tumours [117].

## Novel Cardio-Oncology Biomarkers Under Investigation

### Myeloperoxidase

Myeloperoxidase (MPO) (produced and secreted by leukocytes) has atherogenic and pro-oxidant effects on cardiac tissue leading to its association with increased risk of coronary artery disease and acute HF [118, 119]. MPO is an enzyme produced by polymorphonuclear leukocytes [120], which is pro-atherogenic and pro-oxidant, being responsible for lipid peroxidation, NO scavenging and NO synthase inhibition [121]. In patients in ACS, elevated MPO levels despite normal troponin T levels is important in predicting adverse outcomes [118]. MPO may also be predictive of worse prognosis in acute HF patients [119]and MPO--mediated cardiotoxicityis most likely linked to oxidative stress [122]. MPO has been shown to work synergistically with troponin to predict adverse cardiovascular outcomes. The first description of the link between increase in MPO levels and the development of cardiotoxicity was described following treatment with doxorubicin and trastuzumab [56]. MPO elevation was clinically significant even at baseline (a characteristic unique to this biomarker (*p* = 0.052)) and also clinically significant in terms of the absolute difference in its levels between the second cycle compared to at baseline (HR 1.34; 95%CI, 1–1.8; *p* = 0.048) [56].

### Markers of Extracellular Matrix Turnover and Fibrosis

Galectin-3 could be an important biomarker which plays an important role in remodelling and development of cardiac fibrosis [56, 123]. Galectin-3 is a 26-kDa protein produced by macrophages and stimulates pro-fibrotic pathways leading to fibroblast proliferation and hence collagen deposition. In patients with either acute or chronic decompensated HF, studies have shown Galectin-3 levels are important in predicting mortality rates [124, 125]. ST-2 is a receptor from the Interleukin-33 family and consists of 2 gene forms, i.e. soluble ST2 (sST2) and transmembrane ST2. Blood levels of ST-2 can be considered as less significant predictive factors of mortality and HF [52]. Growth differentiation factor-15 (GDF-15) has anti-apoptosis effects and elevated levels are important in determining prognosis in HF patients [56]. GDF-15 is one of the members of the transforming growth factor beta cytokine family and is released in response to oxidative stress, inflammation and injury [140]. Although GDF-15 levels are elevated in patients with malignancies, it can also be increased in response to oxidative stress, cellular injury and inflammatory stimuli. No association has been made however between elevated levels of GDF-15 or Galectin-3 and development of cardiotoxicity, hence these markers could just be elevated due to malignancy [56].

### MicroRNAs

Non-coding RNAs (microRNAs) mediate cardiac hypertrophy (microRNA-22, microRNA-495 and several more) [126–128] and fibrosis (microRNA-433) [129], and as such may represent important biomarkers of HF. Also, they can be used to differentiate between reduced and preserved ejection HF, as well as determine prognosis. However, both the variability in measurement as well as an incomplete understanding of their pathological role or specificity means that their use is not currently clinically validated [130, 131]. microRNA-146a is important in cardiotoxicity related to doxorubicin and is related to activation of apoptotic pathways by downregulation of ErbB4 [132]. It is considered both a predictive and prognostic indicator, not only in cancer but also in a range of pro-inflammatory conditions ranging from Sjogren’s syndrome to sepsis [133, 134]. This suggests that microRNAs are an important biomarker of cardiotoxicity presenting with inflammation, but more studies are required.

### Insights on Potential New Biomarkers from Genetic Studies

Genome-wide association studies (GWAS) have been instrumental in identifying single nucleotide polymorphisms to identify patients at increased risk of cardiotoxicity due to anthracycline treatment. The largest GWAS involved 1191 patients with HER2-positive breast cancer enrolled in the NCCTG N9831 trial who were treated with anthracyclines and then paclitaxel ± trastuzumab [135]. It has been shown that the maximum reduction of LV function has been associated with the following SNPs in those patients treated with trastuzumab: LDB2 (limb domain binding 2), BRINP1 (BMP/retinoic acid inducible neural-specific protein 1, a suppressor of cell cycle progression), RAB22A (a member of the RAS oncogene family which is key in endocytic trafficking), TRPC6 (transient receptor potential cation channel subfamily C member 6, involved in cardiac remodelling and hypertrophic cardiomyopathy), LINC01060 (a long intergenic noncoding RNA) and chromosome 6 intergenic region. Another GWAS involved a cohort of 385 patients identified targets which have been confirmed to be present in 181 patients [141]. This includes a SNP near PR domain containing 2, with ZNF domain (PRDM2), which is involved in DNA double-strand break and repair of oxidative stress. In this meta-analysis, this was associated with a decline in LVEF (*p* = 6.5 × 10–7). Other GWAS have identified RARG (retinoic acid receptor gamma) which is important in cardiotoxicity and can bind to Top2β (involved in anthracycline-related cardiotoxicity) [142]. In this way, GWAS have been shown to be useful in identifying new SNP loci involved in cardiotoxicity, hence paving the way for the identification of new biomarkers. However, causality cannot be inferred from these studies alone, and no validation studies have been carried out to confirm the findings.

A recent study measured the prevalence of cardiomyopathy causing genes in 3 separate oncology cohorts receiving anthracycline or trastuzumab [143]. In the patients who developed cardiotoxicity with significant new LV dysfunction during or after anthracycline or trastuzumab, there was a significantly higher level of background pathogenic cardiomyopathy causing genetic mutations compared with both matched treated unaffected controls and healthy population (12% vs 2% all-cause mutations) [143]. Truncating mutations of the Titin gene were enriched in the patients with anthracycline or trastuzumab cardiomyopathy compared with a background population (7.5% vs 1.1% cancer controls and 0.7% healthy population). Baseline genetic testing may help identify a subpopulation of cancer patients at higher risk who may be eligible for closer surveillance with cardiac biomarkers during and after treatment with anthracycline or trastuzumab, and also may lead to novel biomarkers for risk stratification and surveillance based upon the specific mutation, e.g. myofilament versus mitochondrial versus apoptotic pathways.

### Proteomics and Metabolomics

In a similar way, proteomic or metabolomic technologies hold great potential in the discovery of new biomarkers linked to cardiotoxicity. Low levels of IgE have been linked to the development of cardiotoxicity [144]. Also instead of mass spectroscopy, newer high throughput screening platforms using aptamers (short single-stranded DNA sequences specific to a particular protein) have been developed, which has helped greatly in the development of this technology as biomarkers [145, 146].

## Biomarker-Guided Cardioprotective Treatment Strategies

In clinical practice, it has been shown that angiotensin-converting inhibitors (ACEI) can halt the progression of ventricular dysfunction as a result of anthracycline treatment [147, 148]. Studies have suggested that the renin-angiotensin-aldosterone system is not directly involved in cardiotoxicity, but may be involved in the remodelling process which takes place afterwards. For example, the International CardioOncology Society-One (ICOS-1) trial investigated whether enalapril started in patients receiving anthracyclines either as primary prevention before and during anthracyclines or as a biomarker-guided cardioprotective strategy where enalapril was started after a detection of a troponin rise [149]. The study concluded that low dose enalapril (mean 5 mg/day) did not reduce anthracycline-related rises in troponin, but it did prevent the related adverse LV remodelling in both study arms.

This was also supported by the PRADA trial where primary prevention with candesartan did not prevent troponin rises in breast cancer cohort receiving anthracycline, but it did reduce adverse remodelling [150]. In contrast, data from both the CECCY [151] and PRADA [150] trials showed that beta blockers prescribed before and during anthracycline treatment could reduce the troponin rise observed in the control arm. However, the beta blocker did not prevent adverse remodelling. Therefore, a combination strategy with beta blockers as primary prevention to prevent myocyte injury and RAAS blocker for patients who develop myocyte injury with troponin elevation is a potential strategy from the results so far.

Similarly, the ongoing Cardiac CARE study (ISRCTN24439460) is designed primarily to assess whether biomarker-guided cardioprotection using treatment with high-dose ACE inhibitors and beta blockers in breast cancer and lymphoma patients where a high-sensitivity troponin assay is used for surveillance [152]. Several studies have shown that a multimodality method involving imaging, as well as biomarkers, is needed. Furthermore, there is evidence to show that the findings are variable depending on whether biomarkers are used pre-, during or post-chemotherapy [46, 74, 95].

## Conclusion

There is evidence to substantiate the use of troponins as a marker of cardiovascular toxicities, such that elevated troponins during anthracyclines are associated with more adverse outcomes and are a significant marker of cardiac dysfunction [46, 74, 149, 153, 154]. Nevertheless, other studies including ICOS-1 [149] have demonstrated that troponin may not always be the most suitable biomarker by demonstrating that it is possible for it to rise independently of cardiotoxicity. On the other hand, other biomarkers including NPs, microRNA, myeloperoxidase and markers of extracellular matrix turnover, are more specific for detecting both acute and chronic effects of anthracyclines and other chemotherapy regimens. In other targeted cancer therapies including trastuzumab, VEGFi and Pl treatment surveillance using NPs has more evidence for detecting subclinical LV dysfunction, predating the development of symptoms and signs consistent with HF. Immune-based cancer therapies may cause myocarditis, and troponin is the marker of choice, but emerging new toxicities including late LV dysfunction mean that NPs may also have a role. This field is open for much research into both novel biomarker discovery and to clarify the exact role of the current cardiac biomarkers (troponin and NPs) in diagnosis, surveillance and biomarker-guided cardioprotective treatment. In this way, the use of combinations of biomarkers will be pivotal towards future strategies of defining the extent of the use of cardioprotective measures.

## References

[1] Dolci A, Panteghini M (2006). The exciting story of cardiac biomarkers: from retrospective detection to gold diagnostic standard for acute myocardial infarction and more. Clinica Chimica Acta.

[2] Zamorano JL, Lancellotti P, Rodriguez MD (2016). 2016 ESC Position Paper on cancer treatments and cardiovascular toxicity developed under the auspices of the ESC Committee for Practice Guidelines. European Heart Journal.

[3] Ponikowski P, Voors AA, Anker SD (2016). 2016 ESC Guidelines for the diagnosis and treatment of acute and chronic heart failure: the Task Force for the diagnosis and treatment of acute and chronic heart failure of the European Society of Cardiology (ESC). Developed with the special contribution of the heart failure association (HFA) of the ESC. European Journal of Heart Failure.

[4] National Cancer Institute. Common Terminology Criteria for Adverse Events (CTCAE).Version 4.0. Available at: http://ctep.cancer.gov/protocolDevelopment/electronic_applications/docs/ctcaev4.pdf. Accessed December 25, 2019.

[5] Curigliano G, Lenihan D, Fradley M (2020). Management of cardiac disease in cancer patients throughout oncological treatment: ESMO consensus recommendations. Annals of Oncology, the journal of the European Society for Medical Oncology.

[6] McGowan JV, Chung R, Maulik A, Piotrowska I, Walker JM, Yellon DM (2017). Anthracycline chemotherapy and cardiotoxicity. Cardiovascular Drugs and Therapy.

[7] Feijen EAM, Leisenring WM, Stratton KL, Ness KK, van der Pal HJH, van Dalen EC, Armstrong GT, Aune GJ, Green DM, Hudson MM, Loonen J, Oeffinger KC, Robison LL, Yasui Y, Kremer LCM, Chow EJ (2019). Derivation of anthracycline and anthraquinone equivalence ratios to doxorubicin for late-onset cardiotoxicity. JAMA Oncology.

[8] Fallah-Rad N, Walker JR, Wassef A (2011). The utility of cardiac biomarkers, tissue velocity and strain imaging, and cardiac magnetic resonance imaging in predicting early left ventricular dysfunction in patients with human epidermal growth factor receptor II–positive breast cancer treated with adjuvant trastuzumab therapy. Journal of the American College of Cardiology.

[9] Pareek N, Cevallos J, Moliner P (2018). Activity and outcomes of a cardio-oncology service in the United Kingdom—a five-year experience. European Journal of Heart Failure.

[10] Moslehi JJ (2016). Cardiovascular toxic effects of targeted cancer therapies. The New England Journal of Medicine.

[11] Henriksen, P. A. (2018). Anthracycline cardiotoxicity: an update on mechanisms, monitoring and prevention. *Heart*, 971–977. 10.1136/heartjnl-2017-312103. 10.1136/heartjnl-2017-312103 29217634

[12] Nowsheen, S., Aziz, K., Park, J. Y., et al. (2018). Trastuzumab in female breast cancer patients with reduced left ventricular ejection fraction. *Journal of the American Heart Association, 7*(15). 10.1161/JAHA.118.008637. 10.1161/JAHA.118.008637 PMC6201446 30371238

[13] Abdel-Rahman O, ElHalawani H, Ahmed H (2015). Risk of selected cardiovascular toxicities in patients with cancer treated with MEK inhibitors: a comparative systematic review and meta-analysis. J Glob Oncol.

[14] Oyakawa, T., Nakashima, K., & Naito, T. (2017). Cardiac dysfunction caused by osimertinib. *Journal of Thoracic Oncology*, e159–e160. 10.1016/j.jtho.2017.05.016. 10.1016/j.jtho.2017.05.016 28939147

[15] O’Hare M, Sharma A, Murphy K, Mookadam F, Lee H (2015). Cardio-oncology part I: chemotherapy and cardiovascular toxicity. Expert Review of Cardiovascular Therapy.

[16] Lyu YL, Kerrigan JE, Lin C-P, Azarova AM, Tsai Y-C, Ban Y, Liu LF (2007). Topoisomerase II mediated DNA double-strand breaks: implications in doxorubicin cardiotoxicity and prevention by dexrazoxane. Cancer Research.

[17] Cardinale D, Colombo A, Bacchiani G (2015). Early detection of anthracycline cardiotoxicity and improvement with heart failure therapy. Circulation.

[18] Shakir D, Rasul KI (2009). Chemotherapy induced cardiomyopathy: pathogenesis, monitoring and management. Journal of Clinical Medical Research.

[19] Odiete O, Hill MF, Sawyer DB (2012). Neuregulin in Cardiovascular Development and Disease. Circulation Research.

[20] Sawyer DB, Zuppinger C, Miller TA, Eppenberger HM, Suter TM (2002). Modulation of anthracycline-induced myofibrillar disarray in rat ventricular myocytes by neuregulin-1beta and anti-erbB2: potential mechanism for trastuzumab-induced cardiotoxicity. Circulation.

[21] Bian Y, Sun M, Silver M (2009). Neuregulin-1 attenuated doxorubicin-induced decrease in cardiac troponins. American Journal of Physiology: Heart and Circulatory Physiology.

[22] Molinaro M, Ameri P, Marone G (2015). Recent advances on pathophysiology, diagnostic and therapeutic insights in cardiac dysfunction induced by antineoplastic drugs. BioMed Research International.

[23] Sawyer DB, Zuppinger C, Miller TA, Eppenberger HM, Suter TM (2002). Modulation of anthracycline-induced myofibrillar disarray in rat ventricular myocytes by neuregulin-1β and anti-erbB2. Circulation.

[24] Maitland ML, Bakris GL, Black HR (2010). Initial assessment, surveillance, and management of blood pressure in patients receiving vascular endothelial growth factor signaling pathway inhibitors. Journal of the National Cancer Institute.

[25] Miller GD, Bruno BJ, Lim CS (2014). Resistant mutations in CML and Ph(+)ALL - role of ponatinib. Biologics.

[26] Scappaticci FA, Skillings JR, Holden SN (2007). Arterial thromboembolic events in patients with metastatic carcinoma treated with chemotherapy and bevacizumab. Journal of the National Cancer Institute.

[27] Choueiri TK, Schutz FAB, Je Y, Rosenberg JE, Bellmunt J (2010). Risk of arterial thromboembolic events with sunitinib and sorafenib: a systematic review and meta-analysis of clinical trials. Journal of Clinical Oncology.

[28] Ghatalia P, Je Y, Kaymakcalan MD, Sonpavde G, Choueiri TK (2015). QTc interval prolongation with vascular endothelial growth factor receptor tyrosine kinase inhibitors. British Journal of Cancer.

[29] Ghatalia P, Morgan CJ, Je Y (2015). Congestive heart failure with vascular endothelial growth factor receptor tyrosine kinase inhibitors. Critical Reviews in Oncology/Hematology.

[30] Schutz FAB, Je Y, Richards CJ, Choueiri TK (2012). Meta-analysis of randomized controlled trials for the incidence and risk of treatment-related mortality in patients with cancer treated with vascular endothelial growth factor tyrosine kinase inhibitors. Journal of Clinical Oncology.

[31] Aparicio-Gallego G, Afonso-Afonso FJ, León-Mateos L (2011). Molecular basis of hypertension side effects induced by sunitinib. Anti-Cancer Drugs.

[32] Eremina V, Jefferson JA, Kowalewska J (2008). VEGF inhibition and renal thrombotic microangiopathy. The New England Journal of Medicine.

[33] Rini BI, Cohen DP, Lu DR (2011). Hypertension as a biomarker of efficacy in patients with metastatic renal cell carcinoma treated with sunitinib. Journal of the National Cancer Institute.

[34] Meyer T, Robles-Carrillo L, Robson T (2009). Bevacizumab immune complexes activate platelets and induce thrombosis in FCGR2A transgenic mice. Journal of Thrombosis and Haemostasis.

[35] Hong S, Fang W, Liang W (2014). Risk of treatment-related deaths with vascular endothelial growth factor receptor tyrosine kinase inhibitors: a meta-analysis of 41 randomized controlled trials. OncoTargets and Therapy.

[36] Hall PS, Harshman LC, Srinivas S, Witteles RM (2013). The frequency and severity of cardiovascular toxicity from targeted therapy in advanced renal cell carcinoma patients. JACC: Heart Failure.

[37] Catino AB, Hubbard RA, Chirinos JA (2018). Longitudinal assessment of vascular function with sunitinib in patients with metastatic renal cell carcinoma. *Circulation*. Heart Failure.

[38] Jain V, Bahia J, Mohebtash M, Barac A (2017). Cardiovascular complications associated with novel cancer immunotherapies. Current Treatment Options in Cardiovascular Medicine.

[39] Touyz RM, Herrmann J (2018). Cardiotoxicity with vascular endothelial growth factor inhibitor therapy. JCO Precision Oncology.

[40] Lenihan D (2017). Cardio-oncology: what is the best practice we can all strive for?. International Journal of Cardiology.

[41] Guha A, Armanious M, Fradley MG (2019). Update on cardio-oncology: novel cancer therapeutics and associated cardiotoxicities. Trends in Cardiovascular Medicine.

[42] Burstein DS, Maude S, Grupp S, Griffis H, Rossano J, Lin K (2018). Cardiac profile of chimeric antigen receptor T cell therapy in children: A single-institution experience. Biology of Blood and Marrow Transplantation.

[43] Alvi RM, Frigault MJ, Fradley MG (2019). Cardiovascular events among adults treated with chimeric antigen receptor T-cells (CAR-T). Journal of the American College of Cardiology.

[44] Plana JC, Galderisi M, Barac A (2014). Expert consensus for multimodality imaging evaluation of adult patients during and after cancer therapy: a report from the American Society of Echocardiography and the European Association of Cardiovascular Imaging. Journal of the American Society of Echocardiography.

[45] Omland T, de Lemos JA, Sabatine MS (2009). A sensitive cardiac troponin T assay in stable coronary artery disease. The New England Journal of Medicine.

[46] Cardinale D, Sandri MT, Martinoni A (2000). Left ventricular dysfunction predicted by early troponin I release after high-dose chemotherapy. Journal of the American College of Cardiology.

[47] Cardinale D, Sandri MT, Colombo A (2004). Prognostic value of troponin I in cardiac risk stratification of cancer patients undergoing high-dose chemotherapy. Circulation.

[48] Cardinale D, Colombo A, Torrisi R (2010). Trastuzumab-induced cardiotoxicity: clinical and prognostic implications of troponin I evaluation. Journal of Clinical Oncology.

[49] Welsh P, Preiss D, Hayward C (2019). Cardiac troponin T and troponin i in the general population: comparing and contrasting their genetic determinants and associations with outcomes. Circulation.

[50] Sawaya H, Sebag IA, Plana JC (2011). Early detection and prediction of cardiotoxicity in chemotherapy-treated patients. The American Journal of Cardiology.

[51] Lipshultz SE, Scully RE, Lipsitz SR (2010). Assessment of dexrazoxane as a cardioprotectant in doxorubicin-treated children with high-risk acute lymphoblastic leukaemia: long-term follow-up of a prospective, randomised, multicentre trial. The Lancet Oncology.

[52] Sawaya H, Sebag IA, Plana JC (2012). Assessment of echocardiography and biomarkers for the extended prediction of cardiotoxicity in patients treated with anthracyclines, taxanes, and trastuzumab. Circulation. Cardiovascular Imaging.

[53] Drafts BC, Twomley KM, D’Agostino R (2013). Low to moderate dose anthracycline-based chemotherapy is associated with early noninvasive imaging evidence of subclinical cardiovascular disease. JACC: Cardiovascular Imaging.

[54] Mornoş C, Petrescu L (2013). Early detection of anthracycline-mediated cardiotoxicity: the value of considering both global longitudinal left ventricular strain and twist. Canadian Journal of Physiology and Pharmacology.

[55] Mavinkurve-Groothuis AMC, Marcus KA, Pourier M (2013). Myocardial 2D strain echocardiography and cardiac biomarkers in children during and shortly after anthracycline therapy for acute lymphoblastic leukaemia (ALL): a prospective study. European Heart Journal Cardiovascular Imaging.

[56] Ky B, Putt M, Sawaya H (2014). Early increases in multiple biomarkers predict subsequent cardiotoxicity in patients with breast cancer treated with doxorubicin, taxanes, and trastuzumab. Journal of the American College of Cardiology.

[57] Putt M, Hahn VS, Januzzi JL (2015). Longitudinal changes in multiple biomarkers are associated with cardiotoxicity in breast cancer patients treated with doxorubicin, taxanes, and trastuzumab. Clinical Chemistry.

[58] Olivieri J, Perna GP, Bocci C (2017). Modern management of anthracycline-induced cardiotoxicity in lymphoma patients: low occurrence of cardiotoxicity with comprehensive assessment and tailored substitution by nonpegylated liposomal doxorubicin. Oncologist.

[59] Kitayama H, Kondo T, Sugiyama J (2017). High-sensitive troponin T assay can predict anthracycline- and trastuzumab-induced cardiotoxicity in breast cancer patients. Breast Cancer.

[60] Shafi A, Siddiqui N, Imtiaz S, Din Sajid MU (2017). Left ventricular systolic dysfunction predicted by early troponin I release after anthracycline based chemotherapy in breast cancer patients. Journal of Ayub Medical College, Abbottabad.

[61] Lipshultz SE, Rifai N, Sallan SE (1997). Predictive value of cardiac troponin T in pediatric patients at risk for myocardial injury. Circulation.

[62] Auner HW, Tinchon C, Linkesch W (2003). Prolonged monitoring of troponin T for the detection of anthracycline cardiotoxicity in adults with hematological malignancies. Annals of Hematology.

[63] Sandri MT, Cardinale D, Zorzino L (2003). Minor increases in plasma troponin I predict decreased left ventricular ejection fraction after high-dose chemotherapy. Clinical Chemistry.

[64] Specchia G, Buquicchio C, Pansini N (2005). Monitoring of cardiac function on the basis of serum troponin I levels in patients with acute leukemia treated with anthracyclines. The Journal of Laboratory and Clinical Medicine.

[65] Kilickap S, Barista I, Akgul E (2005). cTnT can be a useful marker for early detection of anthracycline cardiotoxicity. Annals of Oncology.

[66] Lee HS, Son CB, Shin SH, Kim YS (2008). Clinical correlation between brain natriutetic peptide and anthracyclin-induced cardiac toxicity. Cancer Research and Treatment.

[67] Schmidinger M, Zielinski CC, Vogl UM (2008). Cardiac toxicity of sunitinib and sorafenib in patients with metastatic renal cell carcinoma. Journal of Clinical Oncology.

[68] Ylänen K, Poutanen T, Savukoski T, Eerola A, Vettenranta K (2015). Cardiac biomarkers indicate a need for sensitive cardiac imaging among long-term childhood cancer survivors exposed to anthracyclines. Acta Paediatrica.

[69] Mavinkurve-Groothuis AMC, Groot-Loonen J, Bellersen L (2009). Abnormal NT-pro-BNP levels in asymptomatic long-term survivors of childhood cancer treated with anthracyclines. Pediatric Blood & Cancer.

[70] Pourier MS, Kapusta L, van Gennip A (2015). Values of high sensitive troponin T in long-term survivors of childhood cancer treated with anthracyclines. Clinica Chimica Acta.

[71] Zardavas D, Suter TM, Van Veldhuisen DJ (2017). Role of troponins I and T and N-terminal prohormone of brain natriuretic peptide in monitoring cardiac safety of patients with early-stage human epidermal growth factor receptor 2-positive breast cancer receiving trastuzumab: a herceptin adjuvant study cardiac marker substudy. Journal of Clinical Oncology.

[72] Christenson ES, James T, Agrawal V, Park BH (2015). Use of biomarkers for the assessment of chemotherapy-induced cardiac toxicity. Clinical Biochemistry.

[73] Cardinale D, Biasillo G, Cipolla CM (2016). Curing cancer, saving the heart: a challenge that cardioncology should not miss. Current Cardiology Reports.

[74] Cardinale D, Biasillo G, Salvatici M, Sandri MT, Cipolla CM (2017). Using biomarkers to predict and to prevent cardiotoxicity of cancer therapy. Expert Review of Molecular Diagnostics.

[75] Mulrooney DA, Yeazel MW, Kawashima T, Mertens AC, Mitby P, Stovall M, Donaldson SS, Green DM, Sklar CA, Robison LL, Leisenring WM (2009). Cardiac outcomes in a cohort of adult survivors of childhood and adolescent cancer: retrospective analysis of the Childhood Cancer Survivor Study cohort. BMJ.

[76] Tukenova M, Guibout C, Oberlin O, Doyon F, Mousannif A, Haddy N, Guérin S, Pacquement H, Aouba A, Hawkins M, Winter D, Bourhis J, Lefkopoulos D, Diallo I, de Vathaire F (2010). Role of cancer treatment in long-term overall and cardiovascular mortality after childhood cancer. Journal of Clinical Oncology.

[77] Armstrong GT, Liu Q, Yasui Y, Neglia JP, Leisenring W, Robison LL, Mertens AC (2009). Late mortality among 5-year survivors of childhood cancer: a summary from the childhood cancer survivor study. Journal of Clinical Oncology.

[78] Pardoll DM (2012). The blockade of immune checkpoints in cancer immunotherapy. *Nature Reviews*. Cancer.

[79] Mahmood SS, Fradley MG, Cohen JV (2018). Myocarditis in patients treated with immune checkpoint inhibitors. Journal of the American College of Cardiology.

[80] Bonaca MP, Olenchock BA, Salem JE (2019). Myocarditis in the setting of cancer therapeutics: proposed case definitions for emerging clinical syndromes in cardio-oncology. Circulation.

[81] Lyon, A. R., Yousaf, N., Battisti, N. M. L., Moslehi, J., & Larkin, J. (2018). Immune checkpoint inhibitors and cardiovascular toxicity. *The Lancet Oncology*, e447–e458. 10.1016/S1470-2045(18)30457-1. 10.1016/S1470-2045(18)30457-1 30191849

[82] Johnson DB, Balko JM, Compton ML (2016). Fulminant myocarditis with combination immune checkpoint blockade. The New England Journal of Medicine.

[83] Lee DW, Gardner R, Porter DL, Louis CU, Ahmed N, Jensen M, Grupp SA, Mackall CL (2014). Current concepts in the diagnosis and management of cytokine release syndrome. Blood.

[84] Daugaard G, Lassen U, Bie P (2005). Natriuretic peptides in the monitoring of anthracycline induced reduction in left ventricular ejection fraction. European Journal of Heart Failure.

[85] Lenihan DJ, Massey MR, Baysinger KB (2007). Superior detection of cardiotoxicity during chemotherapy using biomarkers. Journal of Cardiac Failure.

[86] Dodos F, Halbsguth T, Erdmann E, Hoppe UC (2008). Usefulness of myocardial performance index and biochemical markers for early detection of anthracycline-induced cardiotoxicity in adults. Clinical Research in Cardiology.

[87] De Iuliis F, Salerno G, Taglieri L, De Biase L, Lanza R, Cardelli P, Scarpa S (2016). Serum biomarkers evaluation to predict chemotherapy-induced cardiotoxicity in breast cancer patients. Tumor Biology.

[88] Sandri MT, Salvatici M, Cardinale D, Zorzino L, Passerini R, Lentati P, Leon M, Civelli M, Martinelli G, Cipolla CM (2005). N-terminal Pro-B-type natriuretic peptide after high-dose chemotherapy: a marker predictive of cardiac dysfunction?. Clinical Chemistry.

[89] Mukoyama M, Nakao K, Hosoda K (1991). Brain natriuretic peptide as a novel cardiac hormone in humans. Evidence for an exquisite dual natriuretic peptide system, atrial natriuretic peptide and brain natriuretic peptide. Journal of Clinical Investigation.

[90] Kjaer A, Hesse B (2001). Heart failure and neuroendocrine activation: diagnostic, prognostic and therapeutic perspectives. Clinical Physiology.

[91] Romano S, Fratini S, Ricevuto E (2011). Serial measurements of NT-proBNP are predictive of not-high-dose anthracycline cardiotoxicity in breast cancer patients. British Journal of Cancer.

[92] Lenihan DJ, Stevens PL, Massey M (2016). The utility of point-of-care biomarkers to detect cardiotoxicity during anthracycline chemotherapy: a feasibility study. Journal of Cardiac Failure.

[93] Wieshammer S, Dreyhaupt J, Müller D, Momm F, Jakob A (2016). Limitations of N-terminal pro-B-type natriuretic peptide in the diagnosis of heart disease among cancer patients who present with cardiac or pulmonary symptoms. Oncology.

[94] Pavo N, Raderer M, Hülsmann M (2015). Cardiovascular biomarkers in patients with cancer and their association with all-cause mortality. Heart.

[95] Sandri MT, Salvatici M, Cardinale D (2005). N-terminal pro-B-type natriuretic peptide after high-dose chemotherapy: a marker predictive of cardiac dysfunction?. Clinical Chemistry.

[96] Hayakawa H, Komada Y, Hirayama M, Hori H, Ito M, Sakurai M (2001). Plasma levels of natriuretic peptides in relation to doxorubicin-induced cardiotoxicity and cardiac function in children with cancer. Medical and Pediatric Oncology.

[97] Palumbo I, Palumbo B, Fravolini ML (2016). Brain natriuretic peptide as a cardiac marker of transient radiotherapy-related damage in left-sided breast cancer patients: a prospective study. Breast.

[98] Cornell RF, Ky B, Weiss BM (2019). Prospective study of cardiac events during proteasome inhibitor therapy for relapsed multiple myeloma. Journal of Clinical Oncology.

[99] Cowie M (2003). Clinical applications of B-type natriuretic peptide (BNP) testing. European Heart Journal.

[100] Galasko GIW, Lahiri A, Barnes SC, Collinson P, Senior R (2005). What is the normal range for N-terminal pro-brain natriuretic peptide? How well does this normal range screen for cardiovascular disease?. European Heart Journal.

[101] Takase H, Dohi Y (2014). Kidney function crucially affects B-type natriuretic peptide (BNP), N-terminal proBNP and their relationship. European Journal of Clinical Investigation.

[102] Bando S, Soeki T, Matsuura T (2017). Plasma brain natriuretic peptide levels are elevated in patients with cancer. PLoS One.

[103] Mair J, Artner-Dworzak E, Lechleitner P, Smidt J, Wagner I, Dienstl F, Puschendorf B (1991). Cardiac troponin T in diagnosis of acute myocardial infarction. Clinical Chemistry.

[104] Katus HA, Remppis A, Neumann FJ, Scheffold T, Diederich KW, Vinar G, Noe A, Matern G, Kuebler W (1991). Diagnostic efficiency of troponin T measurements in acute myocardial infarction. Circulation.

[105] Cardinale D, Sandri MT, Martinoni A, Borghini E, Civelli M, Lamantia G, Cinieri S, Martinelli G, Fiorentini C, Cipolla CM (2002). Myocardial injury revealed by plasma troponin I in breast cancer treated with high-dose chemotherapy. Annals of Oncology.

[106] Lipshultz SE, Rifai N, Dalton VM, Levy DE, Silverman LB, Lipsitz SR, Colan SD, Asselin BL, Barr RD, Clavell LA, Hurwitz CA, Moghrabi A, Samson Y, Schorin MA, Gelber RD, Sallan SE (2004). The effect of dexrazoxane on myocardial injury in doxorubicin-treated children with acute lymphoblastic leukemia. New England Journal of Medicine.

[107] Rosenberg S., De Vita V., DeVita, L.T. (2015). Hellman, and Rosenberg’s Cancer: Principles & Practice of Oncology.

[108] Libby P (2006). Inflammation and cardiovascular disease mechanisms. The American Journal of Clinical Nutrition.

[109] Hartman J, Frishman WH (2014). Inflammation and atherosclerosis: a review of the role of interleukin-6 in the development of atherosclerosis and the potential for targeted drug therapy. Cardiology in Review.

[110] Ridker PM, Lüscher TF (2014). Anti-inflammatory therapies for cardiovascular disease. European Heart Journal.

[111] Onitilo AA, Engel JM, Stankowski RV, Liang H, Berg RL, Doi SAR (2012). High-sensitivity C-reactive protein (hs-CRP) as a biomarker for trastuzumab-induced cardiotoxicity in HER2-positive early-stage breast cancer: a pilot study. Breast Cancer Research and Treatment.

[112] Lee DW, Gardner R, Porter DL (2014). Current concepts in the diagnosis and management of cytokine release syndrome. Blood.

[113] Frantz S, Falcao-Pires I, Balligand J-L (2018). The innate immune system in chronic cardiomyopathy: a European Society of Cardiology (ESC) scientific statement from the Working Group on Myocardial Function of the ESC. European Journal of Heart Failure.

[114] Zhang Y, Huang Z, Li H (2017). Insights into innate immune signalling in controlling cardiac remodelling. Cardiovascular Research.

[115] Hofmann U, Frantz S (2015). Role of lymphocytes in myocardial injury, healing, and remodeling after myocardial infarction. Circulation Research.

[116] Meng X, Yang J, Dong M (2016). Regulatory T cells in cardiovascular diseases. *Nature Reviews*. Cardiology.

[117] Nahrendorf M, Frantz S, Swirski FK (2015). Imaging systemic inflammatory networks in ischemic heart disease. Journal of the American College of Cardiology.

[118] Baldus S, Heeschen C, Meinertz T (2003). Myeloperoxidase serum levels predict risk in patients with acute coronary syndromes. Circulation.

[119] Reichlin T, Socrates T, Egli P (2010). Use of myeloperoxidase for risk stratification in acute heart failure. Clinical Chemistry.

[120] Hampton MB, Kettle AJ, Winterbourn CC (1998). Inside the neutrophil phagosome: oxidants, myeloperoxidase, and bacterial killing. Blood.

[121] Mukhopadhyay P, Rajesh M, Bátkai S (2009). Role of superoxide, nitric oxide, and peroxynitrite in doxorubicin-induced cell death in vivo and in vitro. *American Journal of Physiology*. Heart and Circulatory Physiology.

[122] Anatoliotakis N, Deftereos S, Bouras G (2013). Myeloperoxidase: expressing inflammation and oxidative stress in cardiovascular disease. Current Topics in Medicinal Chemistry.

[123] van Boxtel W, Bulten BF, Mavinkurve-Groothuis AMC (2015). New biomarkers for early detection of cardiotoxicity after treatment with docetaxel, doxorubicin and cyclophosphamide. Biomarkers.

[124] Chen A, Hou W, Zhang Y, Chen Y, He B (2015). Prognostic value of serum galectin-3 in patients with heart failure: a meta-analysis. International Journal of Cardiology.

[125] Schindler EI, Szymanski JJ, Hock KG, Geltman EM, Scott MG (2016). Short- and long-term biologic variability of galectin-3 and other cardiac biomarkers in patients with stable heart failure and healthy adults. Clinical Chemistry.

[126] Fu J, Chen Y, Li F (2018). Attenuation of MicroRNA-495 derepressed PTEN to effectively protect rat cardiomyocytes from hypertrophy. Cardiol.

[127] Huang ZP, Chen J, Seok HY (2013). MicroRNA-22 regulates cardiac hypertrophy and remodeling in response to stress. Circulation Research.

[128] Colpaert RMW, Calore M (2019). MicroRNAs in cardiac diseases. Cells.

[129] Tao L, Bei Y, Chen P (2016). Crucial role of miR-433 in regulating cardiac fibrosis. Theranostics.

[130] Fichtlscherer S, Zeiher AM, Dimmeler S (2011). Circulating MicroRNAs. Arteriosclerosis, Thrombosis, and Vascular Biology.

[131] Mendell JT, Olson EN (2012). MicroRNAs in stress signaling and human disease. Cell.

[132] Horie T, Ono K, Nishi H (2010). Acute doxorubicin cardiotoxicity is associated with miR-146a-induced inhibition of the neuregulin-ErbB pathway. Cardiovascular Research.

[133] Wang J, Yu M, Yu G (2010). Serum miR-146a and miR-223 as potential new biomarkers for sepsis. Biochemical and Biophysical Research Communications.

[134] Brase JC, Wuttig D, Kuner R, Sültmann H (2010). Serum microRNAs as non-invasive biomarkers for cancer. Molecular Cancer.

[135] Serie DJ, Crook JE, Necela BM (2017). Genome-wide association study of cardiotoxicity in the NCCTG N9831 (Alliance) adjuvant trastuzumab trial. Pharmacogenetics and Genomics.

[136] Haney, S., Cresti, N., Verrill, M., & Plummer, C. J. (2013). Cardiac troponin release following standard dose anthracycline-based adjuvant chemotherapy. *European Heart Journal, 34*(suppl 1), P5752–P5752.

[137] Katsurada K, Ichida M, Sakuragi M, Takehara M, Hozumi Y, Kario K (2014). High-sensitivity troponin T as a marker to predict cardiotoxicity in breast cancer patients with adjuvant trastuzumab therapy. SpringerPlus.

[138] Groenning BA, Nilsson JC, Sondergaard L, Kjaer A, Larsson HBW, Hildebrandt PR (2001). Evaluation of impaired left ventricular ejection fraction and increased dimensions by multiple neurohumoral plasma concentrations. European Journal of Heart Failure.

[139] Morris PG, Chen C, Steingart R, Fleisher M, Lin N, Moy B, Come S, Sugarman S, Abbruzzi A, Lehman R, Patil S, Dickler M, McArthur HL, Winer E, Norton L, Hudis CA, Dang CT (2011). Troponin I and C-reactive protein are commonly detected in patients with breast cancer treated with dose-dense chemotherapy incorporating trastuzumab and lapatinib. Clinical Cancer Research.

[140] Wollert KC, Kempf T, Lagerqvist B, Lindahl B, Olofsson S, Allhoff T, Peter T, Siegbahn A, Venge P, Drexler H, Wallentin L (2007). Growth differentiation factor 15 for risk stratification and selection of an invasive treatment strategy in non–ST-elevation acute coronary syndrome. Circulation.

[141] Wells QS, Veatch OJ, Fessel JP (2017). Genome-wide association and pathway analysis of left ventricular function after anthracycline exposure in adults. Pharmacogenetics and Genomics.

[142] Aminkeng F, Bhavsar AP, Visscher H (2015). A coding variant in RARG confers susceptibility to anthracycline-induced cardiotoxicity in childhood cancer. Nature Genetics.

[143] Garcia-Pavia P, Kim Y, Restrepo-Cordoba MA (2019). Genetic variants associated with cancer therapy-induced cardiomyopathy. Circulation.

[144] Beer LA, Kossenkov AV, Liu Q (2016). Baseline immunoglobulin E levels as a marker of doxorubicin- and trastuzumab-associated cardiac dysfunction. Circulation Research.

[145] Ngo D, Sinha S, Shen D (2016). Aptamer-based proteomic profiling reveals novel candidate biomarkers and pathways in cardiovascular disease. Circulation.

[146] Lind L, Ärnlöv J, Lindahl B, Siegbahn A, Sundström J, Ingelsson E (2015). Use of a proximity extension assay proteomics chip to discover new biomarkers for human atherosclerosis. Atherosclerosis.

[147] Jensen BV, Nielsen SL, Skovsgaard T (1996). Treatment with angiotensin-converting-enzyme inhibitor for epirubicin-induced dilated cardiomyopathy. Lancet.

[148] Okumura K, Jin D, Takai S, Miyazaki M (2002). Beneficial effects of angiotensin-converting enzyme inhibition in adriamycin-induced cardiomyopathy in hamsters. Japanese Journal of Pharmacology.

[149] Cardinale D, Ciceri F, Latini R (2018). Anthracycline-induced cardiotoxicity: a multicenter randomised trial comparing two strategies for guiding prevention with enalapril: The International CardioOncology Society-one trial. European Journal of Cancer.

[150] Gulati, G., Heck, S. L., Røsjø, H., et al. (2017). Neurohormonal blockade and circulating cardiovascular biomarkers during anthracycline therapy in breast cancer patients: results from the PRADA (Prevention of Cardiac Dysfunction During Adjuvant Breast Cancer Therapy) study. *Journal of the American Heart Association, 6*(11). 10.1161/JAHA.117.006513. 10.1161/JAHA.117.006513 PMC5721750 29118031

[151] Avila MS, Ayub-Ferreira SM, de Barros Wanderley MR (2018). Carvedilol for prevention of chemotherapy-related cardiotoxicity: The CECCY trial. Journal of the American College of Cardiology.

[152] ISRCTN24439460. The Cardiac CARE Trial – can heart muscle injury related to chemotherapy be prevented? Http://Www.Who.Int/Trialsearch/Trial2.Aspx?TrialID=ISRCTN24439460. Available at**:** https://www.cochranelibrary.com/central/doi/10.1002/central/CN-01887176/full. Accessed December 22, 2019.

[153] Cardinale D, Cipolla CM (2016). Chemotherapy-induced cardiotoxicity: importance of early detection. Expert Review of Cardiovascular Therapy.

[154] Cardinale D, Sandri MT (2015). Detection and monitoring of cardiotoxicity by using biomarkers: pros and cons: remarks on the international colloquium on cardioncology. Progress in Pediatric Cardiology.

[155] Tocchetti CG, Molinaro M, Angelone T (2015). Nitroso-redox balance and modulation of basal myocardial function: an update from the Italian Society of Cardiovascular Research (SIRC). Current Drug Targets.

[156] Zuppinger C, Suter TM (2010). Cancer therapy-associated cardiotoxicity and signaling in the myocardium. Journal of Cardiovascular Pharmacology.

